# What Fair Dealing Does

**Published:** 1869-07

**Authors:** 


### WHAT FAIR DEALING DOES.

One of the most remarkable cases of legitimate business success which has lately come to our notice is the result, not of a "Fortunate Purchase," nor of a "Real Estate Speculation," nor of a "Wall Street Transaction," but of fair dealing, persisted in. Few colossal fortunes are made in a moment or a month; fortunes thus acquired are always the result of fortunes lost, bringing ruin to two parties; to the loser now, and to the winner a little later; but a fortune is amassing, and if lost, another can be made, "in the same line."

This man has three things in rare proportion.

COURTESY, COURAGE, AND INTEGRITY.

He determined to sell good things at a low price and to let the people know it; always good, always cheap, always cash.

IT PAID,

For fair dealing, low prices, and polite attention please rich and poor alike. Sales increased rapidly; one, three, five hundred dollars a day, then a thousand. Before long, five thousand were realized in a day. We very soon found ourselves looking forward to the report of his Saturday's work with as much interest as the readers of

BONNER'S LEDGER

look to the next publication day of one of his serial stories. Many a Saturday's sales, legitimate, retail, not a dollar credited, have reached six thousand dollars; and on Saturday, May 15, ANNO DOMINI 1869, the footing up of sales of MEN AND BOYS CLOTHING exceeded eight thousand dollars, at the north-east corner of Broadway and Canal street, New York City. ALWAYS GOOD, ALWAYS CHEAP, ALWAYS CASH, and always attentive to his business, are the watchwords of Baldwin the Clothier.

THE GREAT METROPOLIS, a Mirror of New York, being a complete history of metropolitan life and society with sketches of prominent places and persons and things in the city as they actually exist; by Junius Henri Browne; printed at Hartford, Conn., by the American Publishing Company, R. W. Bliss & Co., Toledo, Ohio; Bliss & Co., Newark, N. J. H. H. Bancroft & Co., San Francisco, California. 1869, 700

pp. 8vo., in large clear type and thick white paper; the book can be had only at the places above named or from agents; as it is published by subscription, $5. It has 26 illustrations of the more prominent public places and buildings and ninety chapters treating of the Rich and Poor. New York Society. Wall Street. The Police. The Shipping. The Roughs. First of May. The distinguished women of New York. Greenwood. Parks. Churches. Fifth Avenue. Boarding Houses. Many prominent men. Greeley, Beecher, Raymond, Marble, Stewart, Astor, Vanderbilt, Bennet, &c., &c. It certainly is a most interesting, instructive and valuable book.

